# Perceptions of Overuse Injury Among Swedish Ultramarathon and Marathon Runners: Cross-Sectional Study Based on the Illness Perception Questionnaire Revised (IPQ-R)

**DOI:** 10.3389/fpsyg.2019.02406

**Published:** 2019-10-22

**Authors:** William Wickström, Armin Spreco, Victor Bargoria, Fredrik Elinder, Per-Olof Hansson, Örjan Dahlström, Toomas Timpka

**Affiliations:** ^1^Athletics Research Center, Linköping University, Linköping, Sweden; ^2^Department of Health and Care Development, and Department of Medical and Health Sciences, Linköping University, Linköping, Sweden; ^3^Department of Orthopaedics and Rehabilitation, Moi University, Eldoret, Kenya; ^4^Department of Clinical and Experimental Medicine, Linköping University, Linköping, Sweden; ^5^Department of Management and Engineering, Linköping University, Linköping, Sweden; ^6^Department of Behavioural Sciences and Learning, Linköping University, Linköping, Sweden

**Keywords:** illness perceptions, long-distance running, overuse injury, exercise load, common sense model of illness, sports psychology

## Abstract

**Background:**

Long-distance runners’ understandings of overuse injuries are not well known which decreases the possibilities for prevention. The common sense model (CSM) outlines that runners’ perceptions of a health problem can be described using the categories identity, consequence, timeline, personal control, and cause. The aim of this study was to use the CSM to investigate perceptions of overuse injury among long-distance runners with different exercise loads.

**Methods:**

The study used a cross-sectional design. An adapted version of the illness perception questionnaire revised (IPQ-R) derived from the CSM was used to investigate Swedish ultramarathon and marathon runners’ perceptions of overuse injuries. Cluster analysis was employed for categorizing runners into high and low exercise load categories. A Principal Component Analysis was thereafter used to group variables describing injury causes. Multiple logistic regression methods were finally applied using high exercise load as endpoint variable and CSM items representing perceptions of injury identity, consequence, timeline, personal control, and causes as explanatory variables.

**Results:**

Complete data sets were collected from 165/443 (37.2%) runners. The symptoms most commonly associated with overuse injury were pain (80.1% of the runners), stiff muscles (54.1%), and stiff joints (42.0%). Overuse injury was perceived to be characterized by the possibility of personal control (stated by 78.7% of the runners), treatability (70.4%), and that the injury context was comprehensible (69.3%). The main injury causes highlighted were runner biomechanics (stated by 78.3%), the runner’s personality (72.4%), and running surface biomechanics (70.0%). Among men, a belief in that personality contributes to overuse injury increased the likelihood of belonging to the high exercise load category [Odds ratio (OR) 2.10 (95% Confidence interval (95% CI) 1.38–3.19); *P* = 0.001], while beliefs in that running biomechanics [OR 0.56 (95% CI 0.37–0.85); *P* = 0.006) and mileage (OR 0.72 (95% CI 0.54–0.96); *P* = 0.026] causes injury decreased the likelihood. In women, a strong perception that overuse injuries can be controlled by medical interventions decreased the likelihood of high exercise load [OR 0.68 (95% CI 0.52–0.89); *P* = 0.005].

**Conclusion:**

This study indicates that recognition among long-distance runners of the association between own decisions in overuse injury causation is accentuated by increased exercise loads.

## Introduction

Marathon and ultramarathon running are popular forms of exercise among women and men, and participation in running competitions covering distances longer than 100 km and with 24 h duration continues to increase (Knechtle and Nikolaidis, 2018; Esteve-Lanao et al., 2019; Waldvogel et al., 2019) It is today recognized that overuse injuries constitute a common problem in runners, and that psychological factors play a role in the injury causation (van der Worp et al., 2015; Kerr et al., 2016; Hulme et al., 2017). The opportunity to achieve personal goals has been identified as the main motivation among runners to compete at the longest distances, while runners competing at shorter distances commonly report self-esteem reasons and health-related reasons as equally important (Masters and Ogles, 1995; Ogles and Masters, 2000; Ogled and Masters, 2003). Regarding the psychological effects of long-distance running, already early quantitative research reported an increase in mental fatigue and a decrease in psychological tension, and anxiety (Hassmén and Blomstrand, 1991). These effects were longer lasting than the more short-term mood changes that follow briefer sessions of aerobic exercise (van Wilgen and Verhagen, 2012). As regards overuse injuries, the role of psychological and behavioral factors has been highlighted in qualitative studies (Reed and Ones, 2006; Jelvegård et al., 2016). The results point toward that sportspersons with experiences from this injury type are prone to describe a holistic view on the causal mechanisms, where biological, psychological, and social factors are seen to contribute. Strengthening this multi-factorial view on causation, ultramarathon runners were in a recent experimental study found to have higher cold pain tolerance and lower levels of pain-related anxiety than non-running controls (Roebuck et al., 2018b). The greatest difference in anxiety scores was seen for avoidance behavior, i.e., the runners were mentally less disposed to avoid activities associated with pain. This decreased psychological predisposition to avoid pain among the ultramarathon runners was found to partially mediate the elevated cold pain tolerance.

In order to master the overuse injury problem among long-distance runners, more knowledge of runners’ own understanding of overuse injuries is needed. The common sense model (CSM) of illness (Leventhal et al., 2003) suggests that health problem perceptions can be divided into five main categories: (i) Identity refers to common symptoms of ill health and the extent these are considered to be related to the actual health problem; (ii) The consequence reflect the personal evaluation of the impact of the health problem on personal life; (iii) The timeline reflects the beliefs about the course of the health problem: acute or chronic; (iv) Personal control refers to beliefs about the possibilities for personal control and cure of the health problem; and (v) The cause reflects the beliefs about the causes of the problem. Together with emotional representations, these categories constitute a theoretical foundation for study of approaches to mastering overuse injury strategies also among long-distance runners. To enable comparative studies, Weinman et al. (1996) developed the Illness Perception Questionnaire (IPQ), which was elaborated by Moss-Morris et al. (2002) to the IPQ-R (R for revised). Hagger et al. (2005) introduced the IPQ-R to the sports setting, while van Wilgen et al. (2010) adjusted the instrument to injured sportspersons (IPQ-R-S). The internal consistency of the IPQ-R-S was reported to be adequate for all dimensions and attributions except for the attribution accident or chance.

Even though psychological and behavioral characteristics of long-distance runners have been investigated, several areas with relevance for prevention of overuse injuries among runners with different exercise loads have still not been explored. The aim of this study was to use the CSM to investigate perceptions of overuse injury among long-distance runners with different exercise loads and whether some perceptions distinguish runners with the highest loads.

## Materials and Methods

The study was based on a cross-sectional design. It was performed as a student project at Linköping University. According to Swedish legislation, student projects are not subject to external review by research ethics boards (Etikprövningsmyndigheten, 2019). The study was planned and conducted in accordance with the ethical standards laid down in the 1964 Declaration of Helsinki (6th revision 2008). Informed consent was obtained in writing before interview participation, which was completely voluntary. All study data were handled without breaching the integrity of individual athletes.

### Study Population

The primary study population consisted of all runners listed in the ultramarathon category at the Swedish Athletics Association or as members in the three running clubs in the Stockholm area specialized into ultramarathon and marathon distances. Runner listings with contact information were obtained from the Swedish Athletics Association and the running clubs. The runners were contacted and informed about the study by email.

### IPQ-R-S

The IPQ-R instrument was designed to be adapted to the population to be examined. On the basis of an existing Dutch version adapted to injured athletes (IPQ-R-S) (van Wilgen et al., 2010), a Swedish IPQ-R version (Brink and Alsén, 2017) was adjusted to measure conceptions of overuse injury among active long-distance runners. Throughout the instrument, the word “disease” was replaced by the more specific term “overuse injury” and the wording “my injury” changed to denote “overuse injuries” (in general). The adjusted version (IPQ-R-S-Overuse injury) contains eight dimensions of injury characteristics and five groupings of injury causes.

The first IPQ-R-S-Overuse injury dimension is referred to as “Identity” and asks for perceived specific symptoms and whether these are perceived to be related to overuse injuries. Question 8 was changed from a general symptom (“Red-eyes”) to address a more sports-specific issue (“Too much energy” from IPQ-R-S) and question 23 reworded from “Stiff joints” to “Stiff and/or painful joints.” Two additions were specifically made with regard to the long-distance running context (questions 21 “weight gain” and 24 “stiff and/or painful muscles”).

The remaining seven dimensions of injury characteristics examine the perception of overuse injury by asking to what extent on a five-point scale [1 (absolutely disagree) to 5 (absolutely agree)] the athlete agrees with statements linked to acute/chronic timeline, cyclic timeline, consequences, personal control, treatability (treatment control), the context of ill-health, and emotional representation.

Perceived causes of overuse injury are examined by the 28 in section C of the IPQ-R-S Overuse injury instrument and divided into five subgroups: (i) psychological attributes (for example, the emotional state), (ii) risk factors (for example, previous injury problems), (iii) infectious or immunological causes (for example, infection of virus or bacterium), (iv) accident or coincidence, and (v) a specific subcategory of causes, in this study related to overuse injuries (for example, poor footwear).

### Collection of Data

The data collection was performed using the Briteback Survey Tool TM (Norrköping, Sweden) web-based system in January 2018. A survey was constructed that asked for basic sociodemographic information, exercise load, injury history, and data for the IPQ-R-S Overuse injury. An online version of the survey, an e-mail list, and a mailings schedule were created in the web-based system (Rönnby et al., 2018). The runners were invited to participate in the study through an email that contained study information and a link to the survey. Non-responding runners received maximum two automatic reminders by email with 10-day intervals. Automated system-generated statistics were provided for the researchers immediately after reporting of data.

### Data Analysis

The first step of the data analysis grouped the participating runners with regard to exercise load. A principal component analysis (PCA) was performed to identify different components of the exercise load. The variables used for the analyses were running sessions per week, running hours per week, running miles per week, average long-distance training velocity, hours per week strength training, and hours per week alternative training. Thereafter a cluster analysis was carried out based on the exercise load components to create two (fixed number of clusters chosen as setting) exercise load categories (low, high). Separate cluster analyses were performed for each sex.

In the second step, the runners’ perception of overuse injuries (recognition of, understanding of and perceived main causes of overuse injury) were described according to exercise load categories.

In the third step, a PCA was used to describe perceived compound causal components of overuse injury (variables used: the 28 variables of IPQ-R-S-Overuse-injury Section C).

In the fourth step, binary logistic regression was used to identify aspects associated with high exercise load. The endpoint variable used in the analysis was high exercise load (low/high, as generated from cluster analysis), while the explanatory variables included sex (only in analyses of all participants together), injury history, perceived characteristics of overuse injury (8 variables), and perceived compound causes of overuse injury (components from PCA in step 3). Simple models were first analyzed. Thereafter, all explanatory variables were included multiple models, where the non-significant variables were excluded by Wald’s backward stepwise regression to create separate multiple models for women, men, and the total study population. All analyses were performed in the Statistical Package for the Social Sciences (SPSS version 23).

## Results

### Study Participants

From the primary study population of 443 individuals, data were collected from 165 runners (58 women, 107 men) resulting in a response rate of 37.2%. The average age of the participants was 45.9 years (females 42.3 years, males 47.8 years) (Table 1). About every second runner [43.0% (females 34.0%, males 47.7%)] had suffered a significant injury the previous year (time loss from running at least 3 weeks), and 29.1% (females 37.9%, males 24.3%) had a time loss injury at the time of the study. Also about every second runner [48.6% (females 62.1%, males 41.1%)] used analgesic or anti-inflammatory medication on regular basis.

**TABLE 1:** Inductively created exercise load groups (low load and high load) for female and male long-distance runners determined using cluster analysis.

	**Exercise load groups**								
	
	**Female runners**			**Male runners**			**All runners**		
			
**Runner characteristics**	**Low load *n* = 39**	**High load *n* = 19**	**Total *n* = 58**	**Low load *n* = 66**	**High load *n* = 41**	**Total *n* = 107**	**Low load *n* = 105**	**High load *n* = 60**	**Total *n* = 165**
Age [mean (sd)]	44.0 (9.7)	38.7 (6.8)	42.3 (9.1)	49.6 (9.4)	44.9 (9.8)	47.8 (9.8)	47.6 (9.8)	42.9 (9.3)	45.9 (9.9)
**Main event**									
Half-marathon [*n* (%)]	8 (20.5)	0 (0.0)	8 (13.8)	12 (18.2)	1 (2.4)	13 (12.1)	20 (19.0)	1 (1.7)	21 (12.7)
Marathon [*n* (%)]	27 (69.2)	5 (26.3)	32 (55.2)	51 (77.3)	24 (58.5)	75 (70.1)	78 (74.3)	29 (48.3)	107 (64.8)
Ultra-trail [*n* (%)]	0 (0.0)	1 (5.3)	1 (1.7)	2 (3.0)	3 (7.3)	5 (4.7)	2 (1.9)	4 (6.7)	6 (3.6)
Ultra 6 h/100 km [*n* (%)]	1 (2.6)	4 (21.1)	5 (8.6)	0 (0.0)	3 (7.3)	3 (2.8)	1 (1.0)	7 (11.7)	8 (4.8)
Ultra 12 h/100 miles [*n* (%)]	0 (0.0)	1 (5.3)	1 (1.7)	0 (0.0)	1 (2.4)	1 (0.9)	0 (0.0)	2 (3.3)	2 (1.2)
Ultra 24 h or longer [*n* (%)]	3 (7.7)	8 (42.1)	11 (19.0)	1 (1.5)	9 (22.0)	10 (9.3)	4 (3.8)	17 (28.3)	21 (12.7)
**Exercise**									
Running/week [sessions (sd)]	3.9 (1.0)	6.9 (2.1)	4.9 (2.1)	3.3 (1.0)	6.1 (2.7)	4.4 (2.3)	3.5 (1.0)	6.4 (2.6)	4.5 (2.2)
Running/week [h (sd)]	4.9 (1.5)	10.3 (3.5)	6.7 (3.4)	4.3 (1.5)	7.8 (2.6)	5.6 (2.6)	4.5 (1.6)	8.6 (3.1)	6.0 (3.0)
Running/week [km (sd)]	40.6 (12.9)	89.3 (30.3)	56.5 (30.6)	38.1 (13.3)	82.3 (29.9)	55.0 (30.2)	39.0 (13.2)	84.5 (30.0)	55.6 (30.2)
Running speed (min/km)	5.5 (0.4)	5.3 (0.6)	5.4 (0.5)	5.4 (0.4)	4.9 (0.5)	5.2 (0.5)	5.4 (0.4)	5.0 (0.5)	5.3 (0.5)
Strength training (min/week)	60.8 (34.9)	63.2 (61.8)	61.6 (44.9)	33.2 (29.4)	58.5 (57.1)	42.9 (43.7)	43.4 (34.1)	60.0 (58.1)	49.5 (44.9)
Alternative training [*n* (%)]	29 (74.4)	11 (57.9)	40 (69.0)	42 (63.6)	26 (63.4)	68 (63.6)	71 (67.6)	37 (61.7)	108 (65.5)
Sessions/week (*n*)	1.4 (1.2)	1.9 (2.5)	1.6 (1.7)	1.2 (1.3)	2.0 (2.4)	1.6 (1.8)	1.3 (1.2)	2.0 (2.4)	1.6 (1.8)
**Injury history**									
Previous serious injury [*n* (%)]	11 (28.2)	9 (47.4)	20 (34.5)	32 (48.5)	19 (46.3)	51 (47.7)	43 (41.0)	28 (46.7)	71 (43.0)
Ongoing injury [*n* (%)]	13 (33.3)	9 (47.4)	22 (37.9)	16 (24.2)	10 (24.4)	26 (24.3)	29 (27.6)	19 (31.7)	48 (29.1)
Regular use analgesics [*n* (%)]	22 (56.4)	14 (73.7)	36 (62.1)	23 (34.8)	21 (51.2)	44 (41.1)	45 (42.9)	35 (58.3)	80 (48.5)

### Exercise Load

The principal component analysis based on the six exercise load variables resulted in three components; running quantity (containing the variables running sessions per week, running hours per week, and running miles per week), running speed (average long-distance training velocity), and other exercise practices (hours per week strength training and hours per week alternative training). The cluster analysis based on the three components resulted in 105 athletes being allocated to the low exercise load category and 60 athletes allocated to the high load category (Table 1). Women clustered in the high load category ran more than the women clustered in the low load category in terms of distance (89.3 km vs. 40.6 km per week), time (10.3 h vs. 4.9 h per week), and sessions (6.9 vs. 3.9 sessions per week). Also the male runners clustered in the high load category ran more in terms of distance (82.3 km vs. 38.1 km per week), time (7.8 h vs. 4.3 h per week), and sessions (6.1 vs. 3.3 sessions per week).

### Runners’ Perceptions of Overuse Injury

The recognition of overuse injury among the runners as assessed by the Identity dimension in the IPQ-R-S-Overuse-injury symptoms was diffuse (Table 2). The symptom most commonly reported by the runners (80.1% of the respondents) to be associated with overuse injury was pain. Other symptoms connected with overuse injury were stiff muscles (54.1%), stiff joints (42.0%), and impaired physical ability (40.0%).

**TABLE 2:** Descriptive data for the IPQ-R dimension “Identity” [mean score (standard deviation)] and frequencies of marathon and ultramarathon runners [numbers (percent)] associating IPQ-R symptoms with overuse injury displayed by runner exercise load categories and sex.

		**Training load categories**							
	
	**Female runners**			**Male runners**			**All runners**		
			
	**Low load *n* = 39**	**High load *n* = 19**	**Total *n* = 58**	**Low load *n* = 66**	**High load *n* = 41**	**Total *N* = 107**	**Low load *n* = 105**	**High load *n* = 60**	**Total *n* = 165**
*Identity* [mean (*sd*)]	18.8 (10.0)	24.7 (19.7)	20.6 (13.9)	17.7 (14.3)	19.8 (15.0)	18.5 (14.5)	18.1 (12.8)	21.3 (16.5)	19.2 (14.3)
Pain [*n* (%)]	33 (86.8)	15 (83.3)	48 (85.7)	50 (76.9)	31 (77.5)	81 (77.1)	83 (80.6)	46 (79.3)	129 (80.1)
Stiff muscles [*n* (%)]	27 (69.2)	10 (58.8)	37 (66.1)	31 (49.2)	17 (44.7)	48 (47.5)	58 (56.9)	27 (49.1)	85 (54.1)
Stiff joints [*n* (%)]	19 (48.7)	9 (52.9)	28 (50.0)	23 (35.9)	15 (40.5)	38 (37.6)	42 (40.8)	24 (44.4)	66 (42.0)
Impaired physical ability [*n* (%)]	12 (31.6)	8 (44.4)	20 (35.7)	23 (35.9)	21 (52.5)	44 (42.3)	35 (34.3)	29 (50.0)	64 (40.0)
Restlessness [*n* (%)]	9 (23.7)	4 (22.2)	13 (23.2)	8 (12.5)	12 (30.0)	20 (19.2)	17 (16.7)	16 (27.6)	33 (20.6)
Tiredness [*n* (%)]	4 (10.5)	5 (29.4)	9 (16.4)	12 (19.0)	8 (21.1)	20 (19.8)	16 (15.8)	13 (23.6)	29 (18.6)
Insomnia [*n* (%)]	3 (7.9)	4 (22.2)	7 (12.5)	8 (12.5)	7 (17.5)	15 (14.4)	11 (10.8)	11 (19.0)	22 (13.8)
Upset stomach [*n* (%)]	4 (10.5)	4 (22.2)	8 (14.5)	4 (6.3)	3 (7.5)	7 (6.7)	8 (7.9)	7 (12.1)	15 (9.4)
Breathlessness [*n* (%)]	1 (2.6)	3 (16.7)	4 (7.1)	5 (7.9)	4 (10.3)	9 (8.8)	6 (5.9)	7 (12.3)	13 (8.2)
Weight gain [*n* (%)]	0 (0.0)	3 (16.7)	3 (5.4)	6 (9.4)	2 (5.1)	8 (7.8)	6 (5.9)	5 (8.8)	11 (6.9)
Sore throat [*n* (%)]	1 (2.6)	0 (0.0)	1 (1.8)	1 (1.6)	5 (12.5)	6 (5.8)	2 (2.0)	5 (8.8)	7 (4.4)
Heavy breath [*n* (%)]	0 (0.0)	1 (5.6)	1 (1.8)	3 (4.7)	3 (7.5)	6 (5.8)	3 (2.9)	4 (6.9)	7 (4.4)
Dizziness [*n* (%)]	0 (0.0)	2 (11.8)	2 (3.7)	4 (6.3)	1 (2.6)	5 (4.9)	4 (4.0)	3 (5.4)	7 (4.5)
Headache [*n* (%)]	0 (0.0)	1 (5.6)	1 (1.8)	4 (6.3)	1 (2.5)	5 (4.8)	4 (4.0)	2 (3.4)	6 (3.8)
Nausea [*n* (%)]	3 (7.9)	1 (5.6)	4 (7.1)	1 (1.6)	0 (0.0)	1 (1.0)	4 (3.9)	1 (1.8)	5 (3.1)
Weight loss [*n* (%)]	1 (2.6)	1 (5.9)	2 (3.6)	1 (1.6)	0 (0.0)	1 (1.0)	2 (2.0)	1 (1.8)	3 (1.9)

*The dimension score is standardized (to maximal score 100) using min-max normalization.*

The understanding of overuse injury was among the ultramarathon and marathon runners characterized by possibility to personal control, treatability, and that the injury context was comprehensible (Table 3). Overuse injury was to a lesser extent distinguished by emotional representations and severe consequences for the runner, and the timeline included both cyclic and chronic representation of symptoms.

**TABLE 3:** Descriptive data for the IPQ-R dimensions outlining the perceived characteristics of overuse injury and its perceived causes displayed by exercise load category and sex.

		**Exercise load categories**							
	
	**Female runners**			**Male runners**			**All runners**		
			
**Mean (sd)**	**Low load *n* = 39**	**High load *n* = 19**	**Total *n* = 58**	**Low load *n* = 66**	**High load *n* = 41**	**Total *n* = 107**	**Low load *n* = 105**	**High load *n* = 60**	**Total *n* = 165**
**Overuse injury characteristics**									
Personal control	77.6 (9.5)	77.4 (10.8)	77.5 (9.8)	79.2 (11.7)	79.6 (11.9)	79.4 (11.7)	78.6 (10.9)	78.9 (11.5)	78.7 (11.1)
Treatability	74.0 (13.1)	62.1 (12.8)	70.1 (14.1)	79.2 (12.6)	68.0 (13.5)	70.6 (13.0)	72.9 (12.8)	66.2 (13.4)	70.4 (13.4)
Comprehension of context	71.2 (14.5)	66.6 (19.7)	69.7 (16.3)	71.5 (15.7)	65.1 (17.1)	69.1 (16.5)	71.4 (15.2)	65.6 (17.8)	69.3 (16.2)
Emotional representations	59.5 (17.3)	58.8 (18.2)	59.3 (17.4)	50.6 (17.7)	54.7 (16.7)	52.1 (17.4)	53.9 (18.0)	56.0 (17.1)	54.6 (17.7)
Timeline – cyclic symptoms	51.1 (14.7)	59.2 (9.6)	53.8 (13.7)	52.2 (12.2)	48.9 (15.6)	50.9 (13.6)	51.8 (13.1)	52.2 (14.7)	51.9 (13.7)
Timeline – chronic symptoms	46.9 (15.0)	50.2 (20.6)	48.0 (16.9)	48.4 (16.9)	48.4 (15.7)	48.4 (16.4)	47.9 (16.1)	49.0 (17.3)	48.3 (16.5)
Consequences (for the runner)	46.9 (16.2)	47.1 (14.7)	47.0 (15.6)	42.9 (13.3)	49.8 (15.4)	45.6 (14.5)	44.4 (14.5)	49.0 (15.1)	46.1 (14.9)
Identity	18.8 (10.0)	24.7 (19.7)	20.6 (13.9)	17.7 (14.3)	19.8 (15.0)	18.5 (14.5)	18.1 (12.8)	21.3 (16.5)	19.2 (14.3)
**Overuse injury causes**									
Biomechanics (Runner)	81.4 (13.4)	78.3 (18.1)	80.4 (15.0)	79.5 (13.1)	73.2 (15.9)	77.1 (14.5)	80.2 (13.2)	74.8 (16.7)	78.3 (14.7)
Runner’s personality	69.2 (16.7)	71.7 (18.6)	70.0 (17.2)	70.6 (15.3)	78.7 (14.6)	73.7 (15.4)	70.1 (15.7)	76.5 (16.1)	72.4 (16.1)
Biomechanics (Surface)	74.7 (17.1)	69.1 (16.9)	72.8 (17.1)	68.6 (17.0)	68.3 (16.8)	68.5 (16.9)	70.8 (17.2)	68.5 (16.7)	70.0 (17.0)
Coaching	67.1 (14.3)	65.4 (14.8)	66.5 (14.3)	62.2 (17.3)	58.7 (18.3)	60.9 (17.7)	64.0 (16.4)	60.8 (17.4)	62.9 (16.8)
Exercise overload	63.1 (9.7)	64.1 (11.9)	63.5 (10.4)	63.4 (9.6)	59.3 (9.7)	61.8 (9.8)	63.3 (9.6)	60.8 (10.6)	62.4 (10.0)
Alcohol, smoking, and lifestyle	63.5 (18.8)	61.5 (18.7)	62.8 (18.6)	60.8 (17.2)	60.5 (15.1)	60.7 (16.4)	61.8 (17.8)	60.8 (16.2)	61.4 (17.2)
Predisposition (uncontrollable)	57.1 (16.9)	57.2 (14.0)	57.1 (15.9)	54.7 (15.5)	50.5 (13.4)	53.1 (14.8)	55.6 (16.0)	52.6 (13.9)	54.5 (15.3)
Stress and worry	52.6 (16.5)	55.6 (19.0)	53.6 (17.3)	49.6 (18.5)	51.2 (17.0)	50.2 (17.9)	50.7 (17.8)	52.6 (17.6)	51.4 (17.7)

*The dimension scores are standardized (to maximal score 100) using min-max normalization (standard deviation).*

The runners perceived the main causes of overuse injury to be runner biomechanics, the runner’s personality, and biomechanics associated with the running surface (Table 3). Less dominant causes contributing to overuse injuries were coaching, exercise overload, and alcohol, smoking and lifestyle. Individual predisposition for injury and stress and worry were perceived to be less important causes.

### Compound Components Perceived to Predispose for Overuse Injury

The PCA analysis of the 28 variables in IPQ-R-S-Overuse injury section C resulted in eight causal components perceived by the runners to predispose for overuse injury (Table 4).

**TABLE 4:** Allocation of IPQ-R-S items describing overuse injury causes (C1–C28) into preliminary components (PC1–PC8) and compound causal components (CC1–CC8).

	**IPQ-R-S cause item**	**Preliminary components**								**Causal component (CC)**
			
		**PC1**	**PC2**	**PC3**	**PC4**	**PC5**	**PC6**	**PC7**	**PC8**	
C1	Stress or worry	0.73								CC 1 stress and worry
C4	Diet or eating habits	0.56	0.36							
C9	Mental attitude, e.g., negative thoughts about life	0.75								
C10	Family problems or worries cause overload damage	0.82								
C11	Too much work	0.59						0.38		
C12	Emotional state e.g., depression, loneliness, anxiety, emptiness	0.80								
C18	Changes in the immune system	0.50	0.40							
C14	Alcohol	0.36	0.77							CC 2 alcohol/smoking and lifestyle
C15	Smoking	0.31	0.76							
C16	Accident or injury		0.63							
C23	Poor health status	0.40	0.52					0.32		
C3	A bacterium or virus	0.40		−0.35						CC 3 exercise overload
C7	Environmental pollution			−0.46						
C19	Exercise overload (long continuous period)			0.83						
C20	Exercise overload (acute/short period)			0.79						
C2	Heredity, it is in the family	0.35			0.57					CC 4 predisposition for overuse injury
C5	Coincidences or bad luck				0.77					
C6	Poor past medical care				0.63					
C13	Aging			0.32	0.55					
C26	Coaching/coaches					0.71				CC 5 coaching
C27	Poorly supervised exercise					0.84				
C28	Poor preparation/warm-up		0.35	0.33		0.38	0.36			
C21	Poor/changed equipment			0.37			0.70			CC 6 biomechanics (runner)
C22	Bad material (e.g., shoes)						0.82			
C24	Transition between different surfaces/terrains							0.78		CC 7 biomechanics (surface)
C25	Poor training set-up			0.49				0.52	0.37	
C8	Own behavior			0.32					0.77	CC 8 the runner’s personality
C17	Own personality	0.30			−0.31	0.42			0.47	

*Principal component analysis [correlations in the rotated component matrix (correlation range −1 to 1) are shown].*

Component 1 *Stress and worry* describes the attributes stress and anxiety as explanations for overuse injury. The two variables that most strongly loaded on the component were family problems or concerns followed by the emotional state (depression, loneliness, anxiety, and emptiness).

Component 2 *Alcohol/smoking and lifestyle* describes substance use together with lifestyle as explanations of overuse injury. Alcohol and smoking were the variables with strongest loadings followed by accidents and poor general health status.

Component 3 *Exercise overload* describes exercise load that exceeds the individual’s ability as explanation for overuse injury. The variables with strongest loadings were overtraining (for a longer continuous period) followed by overuse (on occasional occasions).

Component 4 *Predisposition for overuse injury* describes different forms of predisposition or susceptibility. The variables coincidence or bad luck, poor previous medical care, and heredity showed the strongest loadings.

Component 5 *Coaching* includes different aspects of coaching as possible explanation for overuse injury. The strongest loading was shown by poorly monitored exercise.

Component 6 *Biomechanics (runner)* describes various aspects of runner biomechanics. The variable with strongest loading was poor equipment (e.g., shoes) followed by poor running technique.

Component 7 *Biomechanics (surface)* includes different aspects of biomechanics associated with the running surface. The variable with the strongest loading was the transition between different surfaces.

Component 8 *The runner’s personality* includes behavioral items that can explain overuse injury occurrence among long-distance runners. The correlating causal variables were own behavior followed by own personality.

### Differences in Perception Between Runners With High and Low Exercise-Load

In the multiple model for men (Nagelkerke *R^2^* = 0.257), a strong agreement with the statement that personality contributes to overuse injury increased the likelihood of belonging to the high exercise load category [Odds ratio (OR) 2.10 [95% Confidence interval (95% CI) 1.38–3.19]; *P* = 0.001], while a strong perception that running biomechanics [OR 0.56 (95% CI 0.37–0.85); *P* = 0.006] and mileage [OR 0.72 (95% CI 0.54–0.96); *P* = 0.026] contributes to injury causation decreased the likelihood (Table 5). In the multiple model for women (Nagelkerke *R^2^* = 0.229), a strong perception that overuse injuries can be controlled by medical interventions decreased the likelihood of belonging to the higher load category [OR 0.68 (95% CI 0.52–0.89); *P* = 0.005]. In the multiple model for the total study group (Nagelkerke *R^2^* = 0.179), the likelihood of belonging to the higher exercise load category was increased by a strong agreement with that personality contributes to overuse injury [OR 1.57 (95% CI 1.16–2.12); *P* = 0.004] and that overuse injuries have serious consequences [OR 1.11 (95% CI 1.00–1.23); *P* = 0.043]. A strong perception that overload injuries can be controlled by medical interventions decreased the likelihood of belonging to the high-load category [OR 0.77 (95% CI 0.67–0.89); *P* = 0.001].

**TABLE 5:** Associations between perceptions of overuse injury and belonging to the high exercise load category among long-distance runners displayed by sex [odds ratio (95% confidence interval)].

	**High exercise load**					
	
	**Female runners (*n* = 58)**		**Male runners (*n* = 107)**		**All runners (*n* = 165)**	
			
**Overuse injury perceptions**	**Simple models**	**Multiple model**	**Simple models**	**Multiple model**	**Simple models**	**Multiple model**
**Characteristics**						
Treatability	0.68 (0.52–0.89) (*P* = 0.005)	0.68 (0.52–0.89) (*P* = 0.005)	n.s.		0.82 (0.72–0.93) (*P* = 0.003)	0.77 (0.67–0.89) (*P* = 0.001)
Comprehension of context	n.s.		n.s.^∗∗^		0.90 (0.81–0.99) (*P* = 0.031)	
Timeline – cyclic symptoms	1.39 (1.01–1.90) (*P* = 0.041)		n.s.		n.s.	
Severe consequences	n.s.		1.15 (1.02–1.30) (*P* = 0.019)		n.s.^∗^	1.11 (1.00–1.23) (*P* = 0.043)
**Causes**						
Exercise overload	n.s.		0.76 (0.58–0.99) (*P* = 0.040)	0.72 (0.54–0.96) (*P* = 0.026)	n.s.	
Biomechanics (runner)	n.s.		0.67 (0.47–0.96) (*P* = 0.030)	0.56 (0.37–0.85) (*P* = 0.006)	0.72 (0.55–0.96) (*P* = 0.024)	
Runner’s personality	n.s.		1.56 (1.11–2.20) (*P* = 0.011)	2.10 (1.38–3.19) (*P* = 0.001)	1.37 (1.06–1.77) (*P* = 0.016)	1.57 (1.16–2.12) (*P* = 0.004)
*Nagelkerke R^2^*		*R^2^* = 0.229		*R^2^* = 0.257		*R^2^* = 0.179

*^∗^1.09 (1.00–1.20) (*P* = 0.060). ^∗∗^0.89 (0.78–1.00) (*P* = 0.054).*

## Discussion

The aim of this study was to use the CSM to investigate perceptions of overuse injury among ultramarathon and marathon runners and whether some perceptions distinguish runners with the highest loads. Similar to a previous study based on the IPQ-R-S among injured athletes (van Wilgen et al., 2010), we observed that the long-distance runners associated overuse injury with a diffuse illness identity (pain was the outstanding complaint) and a high perceived manageability of the injury problem. Even though the runners related overuse injury with both chronic and cyclic timelines, a high illness coherence suggest that they still perceived they comprehended the nature of this particular threat to their health. The runners expressed trust in their possibilities to manage overuse injuries and generally associated these injuries with moderate consequences in their daily lives. Accordingly, they did not associate the injury category with strong emotional manifestations.

The overuse injury causes brought to the fore by most runners were runner biomechanics, the runner’s personality, and biomechanics associated with the running surface. Previous studies have observed that ultramarathon runners and runners with high exercise loads often are people with a strong drive to explore their physical and mental limits (Masters and Ogles, 1995; Ogles and Masters, 2000; Ogled and Masters, 2003). A question is whether consistent “limits-exploring” traits exist in this category of runners (Roebuck et al., 2018a), and, if so, what impact these traits have on injury predisposition. The male runners with high exercise load in this study were less convinced than those with lower loads that running techniques were decisive for the occurrence of overuse injury. Instead, the importance of personality was highlighted. Also among women, runners with a high exercise load were more prone to indirectly highlight the importance of personal responsibility in prevention of overuse injuries, as they more than runners with a lower load submitted to that possibilities are limited for secondary prevention of overuse injuries using medical interventions. These findings can be compared to previous studies among competitive runners, which have showed that elite runners report “Ignoring pain” as a main risk factor for running injury (Johansen et al., 2017) and that the gradual onset of overuse injuries leads to behavioral responses characterized by neglect of the long-term implications of the injury (Reed and Ones, 2006). Together, these observations suggest that a characteristic of ultramarathon and marathon runners with high exercise loads is that these runners are conscious of their psychological and behavioral response to overuse injury symptoms, in particular pain. Of note, the notions of high exercise load and running experience are not synonymous. There may have been runners who had sustained overuse injuries when rapidly increasing their exercise load, and thereby had gained the insights about their own behavior during a short period of time.

The present findings have some interesting practical implications. The attention observed in this and previous studies to be paid by ultramarathon and experienced marathon runners to their psychological capacity and skills suggest that they are aware of the possibility of being harmed by a deficient inner self-critic. Mindfulness training is therefore an intervention that can relevant to supply to long-distance runners who increase their exercise load. Mindfulness is defined as a non-judgmental, purposeful and moment-to-moment awareness comprising consciousness, awareness, and attention (Kabat-Zinn, 1982). Mindfulness training differs from traditional psychological skills development such as thought control or cognitive reframing by that participants learn to act on situations thoughtfully and with an increased level of awareness and understanding, rather than acting emotionally or impulsively (Chiesa and Serretti, 2009; Lundqvist et al., 2018). It has been reported that a 4-week mindfulness-training intervention among recreational runners resulted in improvements of state mindfulness and trait awareness and decreases in sport-related worries personal standards perfectionism (De Petrillo et al., 2009), while no improvements in actual running performance were found. However, a recent systematic review of the evidence for mindfulness-training approaches in sports showed that although large effect sizes were found for improving mindfulness, flow, and performance, and lower competitive anxiety, none of the 66 studies included were rated as having a low risk of bias (Noetel et al., 2019). Further research using robust designs on mindfulness among ultramarathon and marathon runners on awareness and thoughtful management of pain and overuse injuries is therefore warranted.

This study has strengths and limitations that should be taken into consideration when interpreting the results. Studies on vulnerability in sport are generally scarce despite the topic is included in investigations of resilience and mental toughness (Sarkar and Fletcher, 2014; Uphill and Hemmings, 2016). This study used the an adapted version of the IPQ-R instrument (the IPQ-R-Overuse injury), which provides possibilities for comparisons with other populations of sportspersons suffering from overuse injuries, with perceptions of other health problems in sports, and with illness perceptions in general populations. To facilitate such comparisons, the original terminology and procedures were in the main utilized for the overuse injury adaptation. However, some notions were modified to support interpretations by sports scientists, e.g., the dimension scores were standardized. Moreover, the study population consisted of all competitive ultramarathon and marathon runners listed by the participating organizations. Although the number of invited runners (*n* = 443) was moderate, it represented all competitive long-distance runners in the communities involved. The overall participation rate (37.2%) is comparable to previous studies or slightly lower. Circumstances that affected the participation may have been that the data collection period comprised the pre-season and long weekends (travels abroad, etc.). Nonetheless, due to the limited number of participants, the possibility of type 1 errors occurring in the inference process should be taken into regard. It also should be taken into consideration that the study was performed among runners of Scandinavian decent and having a corresponding cultural and socioeconomic background. Generalization of the results to other populations of long-distance runners should be made with care. Finally, it should be noted that the main competition discipline (ultramarathon, marathon, etc.) was not taken into regard in the clustering of runners into high and exercise low load categories. Having the ambition to compete at ultramarathon distances may thus be associated with other psychological features than those associated with high exercise loads *per se*.

We conclude that the results of this study indicate that recognition among long-distance runners of the association between own decisions and tissue damage in overuse injury causation is accentuated by increased exercise loads.

## Data Availability Statement

The datasets generated for this study are available on request to the corresponding author.

## Ethics Statement

Ethical review and approval was not required for the study on human participants in accordance with the local legislation and institutional requirements. The patients/participants provided their written informed consent to participate in this study.

## Author Contributions

WW, FE, AS, VB, P-OH, ÖD, and TT conceived and designed the research project. WW and TT coordinated the study development, and made substantial contributions to drafting and writing the manuscript. FE and TT recruited the study participants. WW, ÖD, and TT were involved in data collection. AS and ÖD analyzed the data. All authors contributed to the data interpretation and provided a final approval of the version to be published. TT was the guarantor of the integrity of analysis and results.

## Conflict of Interest

The authors declare that the research was conducted in the absence of any commercial or financial relationships that could be construed as a potential conflict of interest.
